# Supplementary figures and images for: Comprehensive assessment of age-specific mortality rate and its incremental changes using a composite measure: A sub-national analysis of rural Indian women
Source: Front Med (Lausanne). 2022 Nov 29;9:1046072. doi: 10.3389/fmed.2022.1046072 (PMC9745315; doi:10.3389/fmed.2022.1046072)

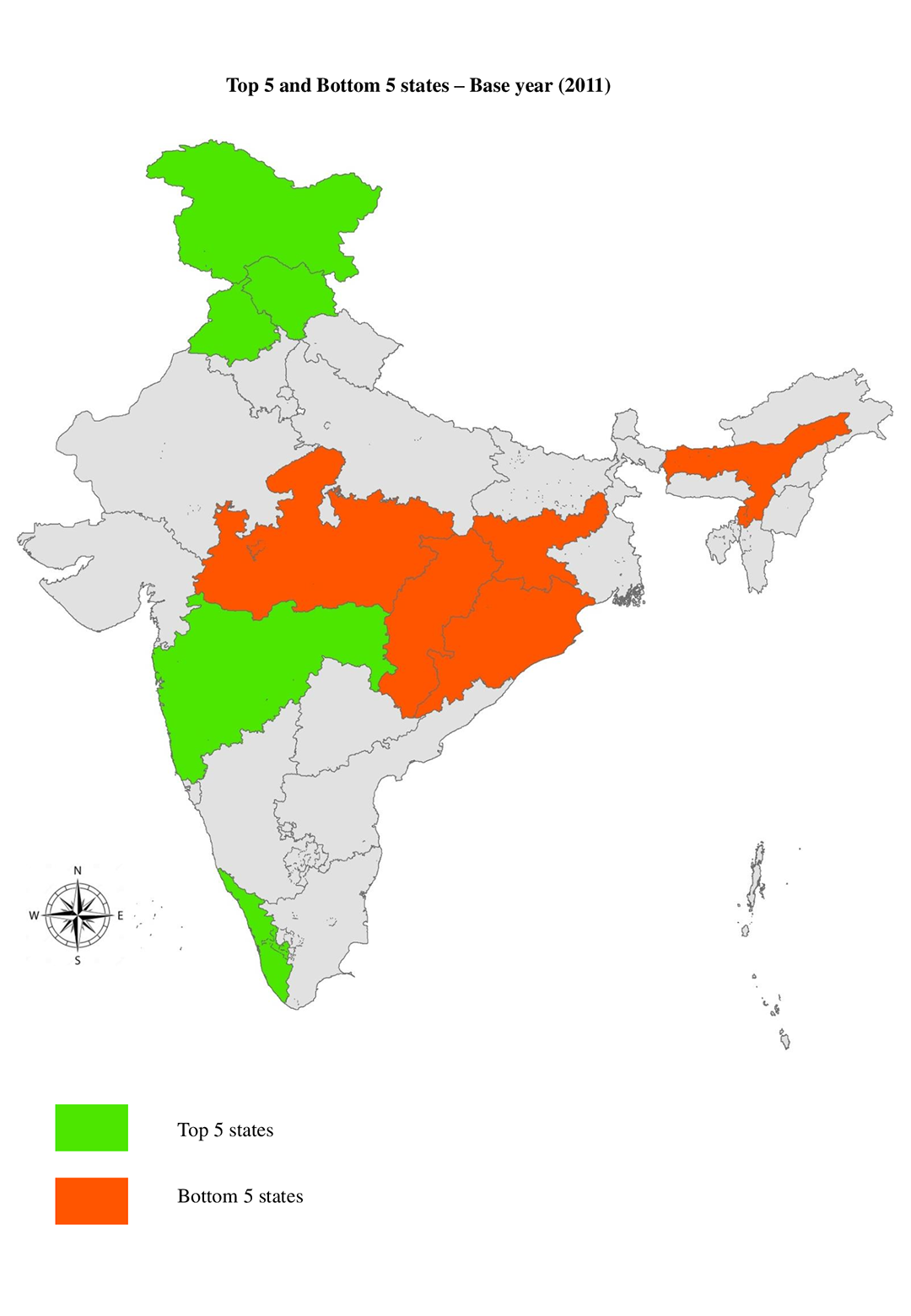

Supplement: Supplementary Figure 1 — Mapping of top five and bottom five states based on composite age-specific mortality index scores in the base year (2011). [file Image_1.TIFF]

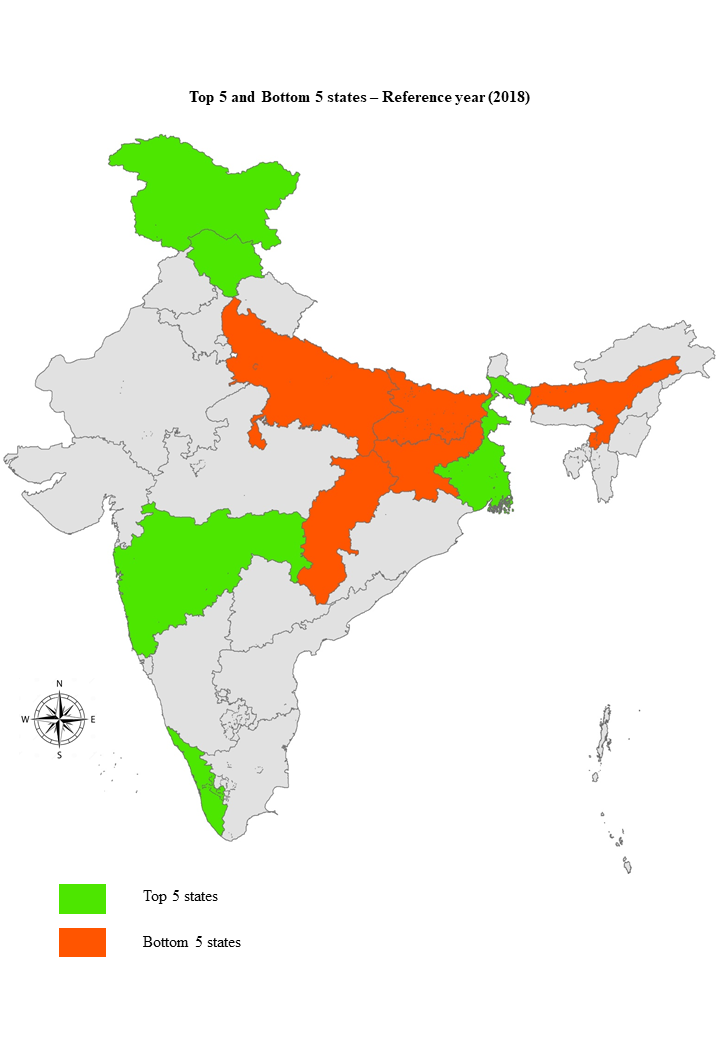

Supplement: Supplementary Figure 2 — Mapping of top five and bottom five states based on composite age-specific mortality index scores in the reference year (2018). [file Image_2.TIF]
