# Supplementary material for: Comprehensive assessment of age-specific mortality rate and its incremental changes using a composite measure: A sub-national analysis of rural Indian women
Source: Front Med (Lausanne). 2022 Nov 29;9:1046072. doi: 10.3389/fmed.2022.1046072 (PMC9745315; doi:10.3389/fmed.2022.1046072)
Supplement: Supplementary file 3 [file Table_1.pdf]

Supplementary Table 1. Minimum and Maximum Age Specific Mortality Rates values of Rural females between 1971 and 2018

S. No	Age group (years)	Rural Female	
		**Maximum value**	**Minimum value**
**1.**	0-4	103.9 (UP, 1976)	1.9 (Kerela, 2001)
**2.**	5-14	6.5 (Assam, 1971)	0.0 (Delhi 2012, 2015-2017)
**3.**	15-59	12.8 (Assam, 1976)	0.6 (Delhi, 2014)
**4.**	60+	87.9 (Assam, 1976)	22.4 (Jammu & Kashmir, 2014)
